# Assessment of health equity consideration in Cochrane systematic reviews and primary studies on urolithiasis

**DOI:** 10.1002/hsr2.1133

**Published:** 2023-02-24

**Authors:** Reyhane Basirat, Sevim Soleimani, Behnam Shakiba, Robab Maghsoudi

**Affiliations:** ^1^ School of Medicine Iran University of Medical Sciences Tehran Iran; ^2^ Student Research Committee, Faculty of Medicine Shahid Beheshti University of Medical Sciences Tehran Iran; ^3^ Department of Urology, Firoozgar Hospital, School of Medicine Iran University of Medical Sciences Tehran Iran; ^4^ Firoozgar Clinical Research Development Center Iran University of Medical Sciences Tehran Iran

**Keywords:** health equity, systematic reviews, urolithiasis

## Abstract

**Background and Aims:**

Health injustice is defined as “unnecessary, preventable, unjustified and unfair health differences.” One of the most important scientific sources on the prevention and management of urolithiasis are Cochrane reviews in this field. Given that the first step in eliminating health injustice is to identify the causes, the aim of the present study was to evaluate equity considerations in Cochrane reviews and the included primary studies on urinary stones.

**Methods:**

Cochrane reviews on kidney stones and ureteral stones were searched through the Cochrane Library. The included clinical trials in each of the reviews published after 2000 were also collected. Two different researchers reviewed all the included Cochrane reviews and primary studies. The researchers reviewed each PROGRESS criteria independently (P: place of residence, R: race/ethnicity/culture, O: occupation, G: gender, R: religion, E: education, S: socioeconomic status, S: social capital and networks). The geographical location of the included studies was categorized as low‐income, middle‐income and high‐income countries, based on the World Bank income criteria. Each PROGRESS dimension was reported for both the Cochrane reviews and the primary studies.

**Results:**

In total, 12 Cochrane reviews and 140 primary studies were included in this study. None of the included Cochrane reviews had specifically mentioned the PROGRESS framework in the Method section whereas gender distribution and place of residence were reported in two and one reviews, respectively. In 134 primary studies at least one item of PROGRESS was reported. The most frequent item was gender distribution, followed by place of residence.

**Conclusion:**

According to the results of this study, the authors of Cochrane systematic reviews on urolithiasis, and researchers who have conducted such trials, have rarely considered health equity dimensions when designing and performing their studies. Therefore, researchers worldwide should be motivated to study populations from low‐income countries with low socioeconomic status in addition to different cultures, ethnicities, and so forth. Furthermore, RCT reporting guidelines such as CONSORT should include health equity dimensions and the editors and reviewers of scientific journals should encourage researchers to further emphasize on health equity in their studies.

## 1 INTRODUCTION

Health advances do not equally benefit all population groups throughout the world; this is verified by the unequal access to resources as well as unequal health situations. These inequalities are not accidental but are exacerbated by social classifying forces.^1^ Social discrimination is a major health issue in both developing and industrialized countries.^2^ There is a significant need for more empirical evidence on efforts made to compensate for the harm caused due to health injustice. Health injustice is defined as “unnecessary, preventable, unjustified and unfair health differences.”^3^ The World Health Organization also defines health injustice as “differences in health status or the distribution of health determinants among different population groups.”^4^ The first step in eliminating health inequity is to identify its causes. In 2003, Evans and Brown introduced the abbreviation “PROGRESS” in this respect. PROGRESS specifically demonstrates the place of residence (e.g., rural, urban, downtown, low‐income countries), Ethnicity/Race/Culture, Language, Occupation, Gender, Religion, Education, Socio‐Economic Level, and Social Capital.^5^ The identification of these factors could provide better opportunities to ensure that the resources are explicitly and measurably allocated to address the inequalities.^1^

On the other hand, urinary stone is a common medical problem with an approximate prevalence of 2% to 3% in the general population. It is estimated that 50% of patients with previous urinary stones experience a recurrence once in 10 years.^6^ This health problem has a major impact on the quality of life and work conditions of these patients and because of the high recurrence rate, it also has a significant impact on the health system. Various authors have identified several factors, including social and demographic variables, leading to urinary stones.^7^ The overall likelihood of developing urinary stones varies depending on several factors such as age, gender, ethnicity, geographical location, climate, and occupation. Men are more likely to have urolithiasis than women.^8^ Moreover, there are socioeconomic differences for most diseases worldwide as Caucasians and Asians are more likely to develop upper urinary stones compared to Hispanics, and African Americans.^9^ Due to these discrepancies in renal stone distribution, to increase the external validity of clinical trials, researchers recommend studying populations which are a true representative of real patients.

One of the most important scientific sources in the prevention and management of urolithiasis are Cochrane reviews. Cochrane reviews are important, high‐quality and practice‐relevant systematic reviews, mainly due to their rigorous, clear and accurate methods and a regular updating process.^10^ They are a valuable source for designing guidelines, policy making and clinical practice.^11^ Given that the first step in eliminating health injustice is to identify the causes, the aim of the present study was to evaluate equity considerations in Cochrane reviews and the included primary studies on urinary stones using the PROGRESS framework.

## 2 METHODS

Cochrane reviews on kidney stones and ureteral stones were searched through the Cochrane Library. The latest search was conducted on June 20, 2022. Two authors (R. B. and S. S.) independently screened all relevant records and the conflict resolution was performed by a third author (B. S.), independently. The included clinical trials in each of the reviews published after 2000 were also collected by reviewing the full text of systematic reviews. We chose this specific time period to better assess health equity in the recent years. There was no limitation in terms of language of publication. Primary studies that were published in full were included and those with only a congress abstract were excluded from our study.

Two different researchers reviewed all the included Cochrane reviews and primary studies. Both researchers collected and recorded information related to the sample size, geographical location of data collection, and the year of publication of each study. These two researchers (S. S. and R. B.) also reviewed each PROGRESS criteria (Table 1) independently based on its guide^1^ for each of the reviews and primary studies using a pretested extraction form. They screened the introduction, method, results and discussion sections for identifying the PROGRESS criteria. In case of presenting supplemental data, the researchers checked these data as well to determine the PROGRESS dimensions. Disagreement was resolved by discussion, and a third author arbitrated if necessary. Geographical location of the included studies was categorized as low‐income, middle‐income and high‐income countries, based on the World Bank income criteria. Each PROGRESS dimension was reported for both Cochrane reviews and the primary studies. The data were analyzed by the Statistical Package for Social Sciences for Windows, version 20 (SPSS Inc).

**Table 1:** different dimensions of health equalities based on PROGRESS framework.

PROGRESS dimensions
Place of residence: Classified as rural, urban, and urban slums; Also includes high‐, middle‐, and low‐income countries. Race/Ethnicity/Culture: Refers to the ethnic, racial, and cultural background as well as the patient's language. Race is predetermined from a biological point of view, while ethnicity and culture are determined by social aspects. Biological differences are not considered unfair unless their manifestation is avoidable. Occupation: Includes a variety of conditions such as disability, part‐time jobs, informal workers, and unsafe work environments. Gender: includes all the social, economic, and cultural characteristics and opportunities, and a social role that is determined for both sexes (based on the phenotype and appearance of the individual). This criterion includes socially conducted rules and other characteristics that society associates with gender. Religion: It is the belief path of each person. This criterion considers inequalities that restrict access to health services for a particular subset of the population with a particular or a lack of any religious orientation. Education: It refers to the degree obtained from valid educational institutions such as schools and universities. It is important because it affects the type of employment and therefore the income of the individual. Educated people are also more aware of health and preventative cares. Socioeconomic status: Measured objectively based on a person's job, education, and income. This factor determines the adequacy of many components that affect health, such as living conditions and access to fresh and healthy food. Social capitals and networks: Include the level of trust between people in a community, civic participation, and the willingness of people in a community to help each other and strengthen their political ties.

Abbreviations: E, education; G, gender; O, occupation; P, place of residence; R, race/ethnicity/culture; R, religion; S, social capital and networks; S, socioeconomic status.

The study protocol was approved by the Ethics Committee of Iran University of Medical Sciences (Ethical approval code: IR.IUMS.FMD.REC.1399.821).

## 3 RESULTS

A total of 12 systematic reviews on urinary stones were included in the present study^12^, ^13^, ^14^, ^15^, ^16^, ^17^, ^18^, ^19^, ^20^, ^21^, ^22^, ^23^ which were published in the Cochrane Database of Systematic Reviews up to January 1, 2022. Details of the included systematic reviews are presented in Table 2.

**Table 2:** Details of the included systematic reviews.

Title	Number of included studies	Sample size	Country of origin
Extracorporeal shock wave lithotripsy (ESWL) versus ureteroscopic management for ureteric calculi	7	1205	HIC
Nonsteroidal anti‐inflammatory drugs (NSAIDs) and nonopioids for acute renal colic	50	5734	HIC
Water for preventing urinary stones	1	220	HIC
Medical and surgical interventions for the treatment of urinary stones in children	14	978	HIC
Alpha‐blockers as medical expulsive therapy for ureteral stones	67	10509	HIC
Medical and dietary interventions for preventing recurrent urinary stones in children	1	125	HIC
Percussion, diuresis and inversion therapy for the passage of lower pole kidney stones following shock wave lithotripsy	2	177	HIC
Alpha‐blockers after shock wave lithotripsy for renal or ureteral stones in adults	40	4793	HIC
Ureteral stent vs. no ureteral stent for ureteroscopy in the management of renal and ureteral calculi	23	1970	HIC
Citrate salts for preventing and treating calcium containing kidney stones in adults	7	477	HIC
ESWL vs. percutaneous nephrolithotomy or retrograde intrarenal surgery for kidney stones	5	338	UMIC
Fluids and diuretics for acute ureteric colic	2	118	HIC

**Figure 1:**
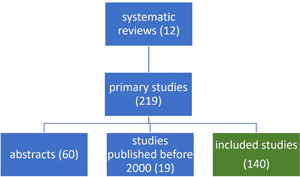
Flow chart of the literature selection process.

These reviews included a total of 219 primary studies. All primary studies published before 2000 and those presented as abstracts only were excluded. Among the remaining 140 primary studies (Figure 1), 6 were not written in English; 5 were originally Korean articles and 1 was an originally Spanish article which were translated into English. We included non‐English language studies with the assistance of translators. In total, 12 Cochrane reviews and 140 primary studies were included in this survey.

## 4 CONSIDERING THE PROGRESS DIMENSIONS IN COCHRANE REVIEWS

None of the included Cochrane reviews had specifically mentioned the PROGRESS framework in the method section whereas gender distribution and place of residence were reported in two and one reviews, respectively (Table 3). Neither of the included reviews had assessed the difference of the intervention effect on any of the PROGRESS items.

**Table 3:** Considering the PROGRESS dimensions in Cochrane reviews and primary studies.

PROGRESS dimensions	Reviews	Primary studies
Place of residence	1 (8.3%)	35 (25%)
Race/ethnicity/culture	0	8 (5.7%)
Occupation	0	0
Gender	2 (16.7%)	130 (92.9%)
Religion	0	0
Education	0	1 (0.7%)
Socioeconomic status	0	0
Social capital and networks	0	0

Abbreviation: PROGRESS, place of residence, race/ethnicity/culture, occupation, gender, religion, education, socioeconomic status, and social capital and networks.

## 5 CONSIDERING PROGRESS DIMENSIONS IN PRIMARY STUDIES

In 134 primary studies at least one item of PROGRESS was reported. The most frequent item was gender distribution, followed by place of residence (Table 3). Similarly, none of the primary studies had reported the difference of the treatment effect on any of the PROGRESS items.

## 6 DISCUSSION

Based on our knowledge, this is the first study trying to ascertain the dimensions of health equity in one of the most important fields of urology by using the PROGRESS equity framework. The decision made to evaluate the Cochrane reviews on urolithiasis was based on two reasons. First, Cochrane reviews are recognized as the top of the pyramid of current evidence hierarchies due to having a standard design and high quality methodology. Second, urolithiasis is a health problem which its' prevalence, incidence and management may be affected by several factors such as age, gender, ethnicity, geographical location, climate, and occupation. Therefore, health equity plays an important role in this respect.

According to the results of this study, the authors of Cochrane systematic reviews on urolithiasis, and researchers who have conducted trials, have rarely considered health equity dimensions when designing and performing their studies. However, most of the studies performed on both male and female patients had considered sex as an individual factor in their analysis. Yet, none of these studies had discussed their findings considering health equity. Although, Cochrane reviews and clinical trials are mostly performed to obtain evidence on the effectiveness of interventions, yet external validity can be strongly increased by using study populations that are similar to real patients.^24^

Evaluation of a total of 152 studies demonstrated that neither had specifically considered the PROGRESS acronym, nor had they pointed to the issue of health equity. This result justifies the great need for the consideration of health equity in Cochrane systematic reviews and their primary studies.

An important characteristic of most of the assessed studies was that they were not conducted in extremely deprived and low‐income countries. This fact reminds us of Horton's “commentary” published by Lancet around 20 years ago.^25^ He stated that there is a “bias against the diseases of poverty” in medical journals in which the studies regarding health problems in the least‐developed regions of the world have the least chance for publication. The present study highlighted other aspects of this bias, mainly that very few studies have been conducted in deprived and low‐income countries.

Therefore, researchers should be motivated to study populations from low‐income countries with low socioeconomic status in addition to different cultures, ethnicities, and so forth. Performing the interventional treatments for urolithiasis among the disadvantaged groups of patients can give researchers a better perspective on the effect of different treatments and can make it possible to effectively compare the outcome of interventions on different races, ethnicities, cultures and religions. This approach could also result in the deprived societies having more access to those interventions.

In addition, RCT reporting guidelines such as CONSORT should include health equity dimensions and editors and reviewers of scientific journals should encourage researchers to further emphasize on health equity in their studies.

Nevertheless, this study suffered from a certain limitation; our search strategy was restricted to the Cochrane library; therefore, we had a limited sample size. Performing similar studies by searching multiple databases can increase the number of retrieved papers leading to higher quality studies.

## 7 CONCLUSIONS

Cochrane reviews and trials on urolithiasis have rarely considered health equity dimensions. Therefore, researchers should be motivated to study populations from low‐income countries with a low socioeconomic status in addition to different cultures, ethnicities, and so forth. RCT reporting guidelines should include health equity dimensions and editors of scientific journals should encourage researchers to further emphasize on health equity in their studies.

## AUTHOR CONTRIBUTIONS

**Reyhane Basirat**: Data curation; methodology; writing—original draft. **Sevim Soleimani**: Data curation; methodology; writing—original draft. **Behnam Shakiba**: Conceptualization; methodology; supervision; writing—review & editing. **Robab Maghsoudi**: Conceptualization; writing—review & editing. All authors have read and approved the final version of the manuscript.

## CONFLICT OF INTEREST STATEMENT

“Behnam Shakiba” is an Editorial Board member of Health Science Reports and a co‐author of this article. To minimize bias, they were excluded from all editorial decision‐makings related to the acceptance of this article for publication. The remaining authors declare no conflict of interest.

## TRANSPARENCY STATEMENT

The lead author Behnam Shakiba affirms that this manuscript is an honest, accurate, and transparent account of the study being reported; that no important aspects of the study have been omitted; and that any discrepancies from the study as planned (and, if relevant, registered) have been explained.

## Data Availability

The authors confirm that the data supporting the findings of this study are available within the article. Behnam Shakiba had full access to all of the data in this study and takes complete responsibility for the integrity of the data and the accuracy of the data analysis.
